# Food Allergy: Psychological Issues—An Editorial Overview

**DOI:** 10.3390/nu18213665

**Published:** 2026-11-04

**Authors:** Laura Polloni

**Affiliations:** 1 Food Allergy Referral Centre of Veneto Region, Department of Women’s and Children’s Health, Padua University Hospital, 35128 Padua, Italy; laura.polloni@aopd.veneto.it; 2 Unit of Psychology, Padua University Hospital, 35128 Padua, Italy

Food allergy (FA) is not simply an immunological reaction to specific proteins; it is a profound chronic condition that redefines the relationship between the patient and their caregiver with their environment, their community, and nutrition itself. In recent decades, an increasing number of studies have shown that FA is associated not only with an increased risk of nutritional deficiencies and growth delays, but also with wide-ranging psychological problems.

Adhering to a strict allergen-free diet impairs health-related quality of life across all age groups [1,2,3]. Navigating social situations and daily food choices imposes a heavy emotional burden characterized by constant vigilance [4]. Furthermore, acute, life-threatening reactions increase mental health risks for children and parents, compromising their relationship with safe food [5]. Recent global studies confirm this significant psychological distress in adults, children, and caregivers [6,7].

These findings are in line with the European Academy of Allergy and Clinical Immunology’s clinical guidelines which officially recognize the significant psychological impact of FA and recommend the inclusion of routine psychological assessment, alongside nutritional counseling, in allergy treatment protocols [8].

This Special Issue entitled “Food Allergy: Psychological Aspects” offers a timely and much-needed analysis of this field. Ranging from qualitative maternal narratives to preliminary adult behavioral data, these five papers highlight the complex interplay between strict dietary management and psychological vulnerability, offering an integrated framework for the modern management of FA.

In DunnGalvin’s comprehensive review [Contribution 1], FA is analyzed through a rigorous biopsychosocial model that considers the intersection between biology, neuropsychology, and social dynamics. FA acts as a chronic stressor that alters the neurobiological systems.

The constant unpredictability of accidental exposure permanently activates threat-detection circuits and impairs dopaminergic reward pathways. Consequently, patients often experience interoceptive distortions—misinterpreting benign bodily sensations as imminent anaphylaxis—which fuels anxiety and social isolation. Additionally, fear-driven avoidance generalizes over time, while strict diets strip eating of its psychological and social reward value. The author concludes that treating FA solely as an immune response ignores these profound neurobiological impacts, highlighting the urgent need for multidisciplinary care.

The consequences of this ongoing vigilance become particularly apparent when patients reach adulthood. Whilst pediatric FA has been investigated, data on adults have remained scarce. Two groundbreaking studies published in this Special Issue directly address this gap, demonstrating a worrying link between necessary dietary restrictions and the development of eating disorders (ED).

Hine et al. [Contribution 2] explored the prevalence of ED screening and associated factors among adults. FA alone was not statistically associated with current positive screening but was linked to ED lifetime history and treatment, suggesting that this relationship may be more evident earlier in the FA experience. The authors hypothesize that a recent FA diagnosis can trigger a conditioned fear model, where unpredictable reactions cause anxiety and hypervigilance. This distress may lead to avoidant and restrictive eating behaviors that gradually subside as patients learn to recognize safe foods and the reactions become less frequent. Although further longitudinal research is needed to clarify when and how FA experience is linked to ED, these preliminary findings highlight the importance of a multidisciplinary approach that integrates routine psychological screening into allergy management to detect FA distress and related eating difficulties early.

Complementing this, Polloni et al. [Contribution 3] provided preliminary research on Avoidant/Restrictive Food Intake Disorder (ARFID) and food neophobia (FN) in adult patients, revealing that 73% screened positive for FN and 25% for ARFID and proving these disorders are not limited to childhood. In adults, the constant fear of contamination and allergic reactions can lead to a gradual, arbitrary restriction of “safe” foods far beyond medical requirements. This allergy-specific anxiety and restrictive eating pattern significantly increase the likelihood of developing both disorders. The study highlights how a chronically monotonous diet can expose patients to specific micronutrient deficiencies while triggering psychological difficulties that further worsen their nutrition and overall health. Ultimately, clinicians should recognize the risk of ARFID and FN in adult patients and recommend appropriate psychological and dietary support.

The psychological burden of FA is rarely confined to the individual patient; it ripples outward, fundamentally altering the dynamics of the whole family. The remaining articles in this Special Issue focus on caregivers, who have to bear the logistical and emotional burden of managing elimination diets and ensuring their children’s safety.

In their qualitative study, Harbottle et al. [Contribution 4] explored unmet needs relating to cow’s milk protein allergy amongst pregnant women and mothers of infants/toddlers. The participants described a profound sense of isolation, psychosocial distress and frustration with the healthcare system. Many mothers reported initially feeling belittled or ignored by clinicians, and described access to allergy specialists as difficult and slowed down by long waiting lists. This gap in care drives parents to seek answers online themselves—a process that often fuels anxiety through conflicting and overwhelming information. The authors conclude that managing FA in early childhood entails financial, social and psychological burdens that current medical protocols largely fail to address.

This burden scales up when a child suffers from multiple sensitivities. Cay et al. [Contribution 5] utilized data from the FORWARD (Food Allergy Outcomes Research and Education Programme) study to estimate the psychosocial burden associated with increasing numbers of current FA among children with multi-FA and their caregivers. The findings reveal that this burden increases monotonically with each additional FA. While managing FA is universally demanding, the intensity and ubiquity of daily vigilance impose a significantly heavier psychosocial burden on families dealing with multiple FA. Crucially, this linear trend remains independent of demographic variables and socioeconomic status, highlighting how the fear of fatal outcomes universally affects families. Consequently, the study emphasizes the clinical necessity of integrating screening and psychological support into standard pediatric allergy management protocols.

In conclusion, the articles in this Special Issue of *Nutrients* share a common conclusion: FA management requires a multidisciplinary approach. Prescribing an elimination diet does not merely alter food intake; it changes the patient’s neurobiology, introduces potential triggers for mental health disorders, and imposes heavy emotional and financial burdens on families. Therefore, clinicians must proactively identify psychosocial distress during routine care. By providing integrated immunological, nutritional, and psychological care, we can enhance patient and family well-being, encourage effective management strategies, and prevent further complications and comorbidities.

As Guest Editor of this Special Issue, I express my deepest gratitude to all the authors for contributing their high-quality research and advancing our understanding of the psychological dimensions of food allergies. I also sincerely thank the reviewers for their rigorous evaluation of the manuscripts, and the *Nutrients* editorial team for their continuous support.
